# Transcriptomic profile of lettuce seedlings (*Lactuca sativa*) response to microalgae extracts used as biostimulant agents

**DOI:** 10.1093/aobpla/plad043

**Published:** 2023-07-02

**Authors:** Danilo F Santoro, Ivana Puglisi, Angelo Sicilia, Andrea Baglieri, Emanuele La Bella, Angela Roberta Lo Piero

**Affiliations:** Department of Agriculture, Food and Environment, University of Catania, via Santa Sofia 98, 95123 Catania, Italy; Department of Agriculture, Food and Environment, University of Catania, via Santa Sofia 98, 95123 Catania, Italy; Department of Agriculture, Food and Environment, University of Catania, via Santa Sofia 98, 95123 Catania, Italy; Department of Agriculture, Food and Environment, University of Catania, via Santa Sofia 98, 95123 Catania, Italy; Department of Agriculture, Food and Environment, University of Catania, via Santa Sofia 98, 95123 Catania, Italy; Department of Agriculture, Food and Environment, University of Catania, via Santa Sofia 98, 95123 Catania, Italy

**Keywords:** Biostimulant, Chlorella vulgaris, lettuce, microalgae, RNAseq, Scenedesmus quadricauda

## Abstract

To reduce the use of chemical fertilizers and maximize agricultural yields, the use of microalgae extracts as biostimulants has recently attracted significant attention due to their favourable impact on both plant growth and their ability to induce tolerance towards environmental stressors. Lettuce (*Lactuca sativa*) is one of the most important fresh vegetables that often requires applications of chemical fertilizers to increase quality and productivity. Accordingly, the purpose of this study was to analyse the transcriptome reprogramming of lettuce (*L. sativa*) seedlings in response to either *Chlorella vulgaris* or *Scenedesmus quadricauda* extracts by applying an RNAseq approach. Differential gene expression analysis revealed that the core gene set that responded to microalgal treatments in a species-independent manner includes 1330 clusters, 1184 of which were down-regulated and 146 up-regulated, clearly suggesting that the repression of gene expression is the main effect of algal treatments. The deregulation of 7197 transcripts in the *C. vulgaris* treated seedlings compared to control samples (*Ls*Cv vs. *Ls*CK) and 7118 transcripts in the *S. quadricauda* treated seedlings compared to control samples (*Ls*Sq vs. *Ls*CK) were counted. Although the number of deregulated genes turned out to be similar between the algal treatments, the level of deregulation was higher in *Ls*Cv versus *Ls*CK than in *Ls*Sq versus *Ls*CK. In addition, 2439 deregulated transcripts were observed in the *C. vulgaris* treated seedlings compared to *S. quadricauda* treated samples (*Ls*Cv vs. *Ls*Sq comparison) suggesting that a specific transcriptomic profile was induced by the single algal extracts. ‘Plant hormone signal transduction’ category includes a very elevated number of DEGs, many of them specifically indicating that *C. vulgaris* actives both genes involved in the auxin biosynthesis and transduction pathways, whereas *S. quadricauda* up-regulates genes implicated in the cytokinin biosynthesis pathway. Finally, algal treatments induced the deregulation of genes encoding small hormone-like molecules that are known to act alone or by interacting with major plant hormones. In conclusion, this study offers the groundwork to draw up a list of putative gene targets with the aim of lettuce genetic improvement that will allow a limited or even null use of synthetic fertilizers and pesticides in the management of this crop.

## Teaser

A crucial challenge is to meet food demands along with improving agricultural sustainability. The use of microalgae extracts as biostimulants has recently attracted significant attention due to their favourable impact on crop growth, yields, quality, nutrient uptake and tolerance to abiotic stresses. In this work, the transcriptomic profile of lettuce seedling subjected to either *Chlorella vulgaris* or *Scenedesmus quadricauda* treatments was analysed taking into account that lettuce is one of the most important fresh vegetables that often requires applications of chemical fertilizers. Among the two microalgae extracts, *C. vulgaris* induced a qualitatively and quantitatively deeper response than that obtained using *S. quadricauda* extract, although both treatments led to ameliorated morpho-biometric parameters. ‘Phytohormones’ is the category including several differentially expressed genes showing that *C. vulgaris* activates both the auxin biosynthesis and transduction pathways whereas *S. quadricauda* up-regulates the cytokinin biosynthesis pathway, probably because they are rich in different amounts and types of beneficial components.

## Introduction

Over the past few years, several studies have been conducted to quantify the impact of climate change on crop productivity (Asseng *et al.* 2015; Webber *et al.* 2015; Zhao *et al.* 2015; Bennici *et al.* 2019). Considering that it has been reported that the human population might grow to reach 9.6 billion by 2050 (Bruinsma 2009), emerging breakthroughs are needed to increase crop productivity worldwide and to meet the human requirements in terms of food supplies. From an organic agricultural point of view, the use of chemical fertilizers and pesticides should be limited (Pascual *et al.* 2018) as they pose unsolved issues to human health and the environment. Consequently, the necessitates for new eco-sustainable organic compounds have arisen to reduce the dependency on agrochemical compounds which is typical of the conventional agricultural practice. Plant biostimulants are gaining an increasing attention to address environment-friendly crop management due to their positive effects on plant growth (Martínez-Viveros *et al.* 2010; Bhattacharyya and Jha 2012), resulting in enhanced nutrient use efficiency, tolerance to abiotic stresses, and improved crop quality and yield (Drobek *et al.* 2019). Microalgae are photosynthetic, autotrophic, or heterotrophic unicellular micro-organisms which are mostly found in freshwater and marine environments. Among the wide range of biostimulant resources (Abbott *et al.* 2018), microalgae and their extracts have been shown to positively influence plant physiology by affecting both the transcriptomic and metabolomic patterns of the treated plants (Jannin *et al.* 2013; Battacharyya *et al.* 2015; Ali *et al.* 2022) acting either on the plant primary metabolism or secondary metabolism pathways and leading to a generalized increase of plant fitness (Franzoni *et al.* 2022). Notably, these compounds can be added to the soil in small quantities (Bulgari *et al.* 2015) provoking an enhancement of water uptake, root and shoot growth, tolerance to abiotic stress conditions, protein content, and the activity of several enzymes related to nitrogen assimilation and photosynthesis processes (Parrado *et al.* 2008; Baglieri *et al.* 2014). The effectiveness of biostimulants on plant physiology is not due to single components of extracts but depends on the synergistic action of different bioactive molecules (Rouphael and Colla 2018) including polysaccharides, phenolics, fatty acids, vitamins, osmolytes and phytohormones (Franzoni *et al.* 2022). Moreover, the quantity and quality of biologically active metabolites in microalgal extracts largely depend on the species used and on the extraction technique (Puglisi *et al.* 2018). The characterization of the biostimulant action of *Chlorella vulgaris* and *Scenedesmus quadricauda* microalgae extracts has been carried out on several crops such as sugar beet (Barone *et al.* 2018) and tomato (Barone *et al.* 2019) registering in both cases a sharp enhancement of shoot and root dry and fresh weight (FW; Barone *et al.* 2018, 2019).

*Lactuca sativa* is one of the major horticulture crops grown in the Mediterranean basin, which often requires the use of chemical fertilizers to reach high levels of productivity also being a moderately salt-sensitive crop (Lucini *et al.* 2015). Recently, the effect of either *C. vulgaris* or *S. quadricauda* extracts on lettuce seedling growth was investigated (Puglisi *et al.* 2020a, 2022). The results showed that both algal extracts ameliorated seedling growth by promoting an increase in dry matter, in photosynthetic pigment content, and inducing the activities of several enzymes involved in primary and secondary metabolism (Puglisi *et al.* 2020a, 2022). Similarly, a formulation based on *C. vulgaris* extract combined with plant growth-promoting bacteria was also evaluated revealing a positive effect on the yield and nutritional parameters, on the total antioxidant activity as well as on the carotenoid content in romaine lettuce leaf (Kopta *et al.* 2018).

The characterization of the global molecular mechanisms by which microalgae extracts exert their effects on plants can be obtained using *-omics* approaches. Transcriptomic analysis based on Next-Generation Sequencing made the development of genomic resources progressively simpler and cheaper. It represents one of the most powerful tools allowing the quantitative determination of all the virtually expressed genes in a specific organ, as well as of the biological processes and metabolic pathways deregulated in response to an external stimulus (Sicilia *et al.* 2019, 2020; Russo *et al.* 2021). *De novo* transcriptome analysis has been also applied in lettuce to identify genes specifically induced by UV-B radiation (Zhang *et al.* 2019) or by inoculation with the necrotrophic fungus *Botrytis cinerea* (de Cremer *et al.* 2013). More recently, the transcriptomic profiles of young and old leaves of lettuce grown under different light sources were also unravelled to identify the optimal illumination conditions for green-vegetable production (Nagano *et al.* 2022).

Taking into account both the acquired knowledge regarding the stimulating effects of microalgae extracts on lettuce seedling’s growth (Puglisi *et al.* 2020, 2022) and the worldwide increasing interest in biofertilizers, the objective of this work was to shed light upon the effects of *C. vulgaris* and *S. quadricauda* extracts on lettuce seedling transcriptomic profile. As far as we know, this is the first report on the global transcriptomic analysis of lettuce leaves treated with algal extracts.

## Material and Methods

### Microalgae culture and extract preparation

The microalgae used in this study were *C. vulgaris* (Beijerinck, CCAP 211/11C) and *S. quadricauda* (isolated from an algal company raceway pond, located in Borculo, Gelderland, the Netherlands, in 2011). They were obtained and maintained in the algal collection of the Department of Agriculture, Food and Environment (Di3A) of University of Catania. Microalgal growth was conducted in 250-mL flasks containing 150 mL of sterile standard BG11 algae medium (Stanier *et al.* 1971) at pH 8.4, incubated on a mechanical shaker (100 rpm) at 25–30 °C, bubbled with air and illuminated by a 3500-lux, average photon flux (PPF) 100 µmol m^−2^ s^−1^ light source (PHILIPS SON-T AGRO 400) with a 12-h photoperiod for 30 days in a growth chamber and aerated by pumps with 20 L h^−1^ 1.5 % CO_2_. Microalgal biomasses were harvested by centrifugation at 5000 rpm for 15 min, then the pellet was washed several times with distilled water to reach a conductivity <200 µS cm^−1^ and finally freeze-dried as described by Puglisi *et al.* (2018, 2019). Microalgae extract stock solutions were prepared as described by Barone *et al.* (2018). Briefly, microalgae cells were centrifuged at 5000 rpm for 15 min and methanol was added (1:12 w/v ratio) to the final pellet. The mixture was mechanically shaken overnight to disperse the biomass in the solvent system, lyse the cell wall and obtain the intracellular extracts. Then, the organic solvent was removed through centrifugation at 5000 rpm for 15 min and evaporation via rotary vapour. Finally, the extracts were freeze-dried and collected with distilled water to obtain the extract of microalgae stock solution as reported in Puglisi *et al.* (2020*a*). The characterization of the biomass of *C. vulgaris* and *S. quadricauda* and their extracts are reported in detail in Barone *et al.* (2018).

### Experimental conditions

The experiment was conducted in transparent boxes (40 × 20 × 10 cm), containing pumice as an inert substrate wetted with 1 L of Hoagland solution (Arnon and Hoagland 1940) as detailed in (Puglisi *et al.* 2020*b*). Lettuce seedlings (*Lactuca sativa*) were provided by a local nursery and 10 seedlings at ‘four true leaves’ stage were transplanted in each box in a completely random design, performing five biological replicates for treatments. The seedlings were grown for 6 days in a growth chamber at 25 ± 2 °C, with a 16-h photoperiod and they were irrigated every day with 100 mL distilled water. After this period of acclimatization (6 days), the treatment was performed by irrigating the inert substrate with Hoagland solution (500 mL) containing either *C. vulgaris* (*Ls*Cv sample) or *S. quadricauda* (*Ls*Sq sample) extracts at the concentration of 1 mg of organic carbon per litre (Corg L−^1^), whereas the untreated plants (*Ls*CK) received only 500 mL of Hoagland solution (Puglisi *et al.* 2020*b*). Leaf tissue was collected both in treated (*C. vulgaris* and *S. quadricauda*) and untreated plants after 4 days from the treatment and immediately frozen in liquid nitrogen and stored at −80 °C until further use (sampling T4[I]).

### Morpho-biometric parameters in lettuce seedlings

Lettuce seedlings were collected, separated into roots and shoots, and the FW of leaves and roots was separately determined (0.01 g accuracy). The dry weight (DW) was obtained by placing a set of subsample tissue in a drying oven at 105 °C until constant weight, and, after allowing to cool for 2 h inside a closed bell jar, the DW was recorded. For each sample, the Relative Growth Rate (RGR) index was also determined. It represents the relative increase in weight per day, calculated according to the following equation (Gent, 2017): RGR = [ln(weight2) − ln(weight1)]/(t2 − t1), where weight2 and weight1 represent the DW at the sampling time [sampling T4(I)] and the FW at the beginning of the experimental period, respectively; t2 and t1 represent the end and the initial time of the experimental period (11 and 0 days, respectively). Statistical analysis was performed by evaluating the effects of single factor on lettuce seedlings by using Minitab (version 16.1.1, Minitab Inc., State College, PA) by one-way ANOVA (*P* < 0.05). The arithmetic mean of each parameter was calculated by averaging the values of ratios and RGR determined for the single replicates of each treatment. Post-hoc analysis was performed by Fisher’s least significant difference test (*P* = 0.05). The biochemical characterization of seedling samples used in the following transcriptome analysis is reported in Puglisi *et al.* (2020*b*) and Puglisi *et al.* (2022) [sampling T4(I)], and includes protein and pigment content, as well as several enzyme activities involved in primary (carbon and nitrogen) and secondary metabolism.

### Sample collection and RNA extraction

RNA isolation was carried out by using the Spectrum Plant Total RNA Extraction kit (Sigma-Aldrich, St. Louis, MO) according to the manufacturer’s instructions (Santoro *et al.* 2022). RNA purity and concentration were assayed using the NanoDrop spectrophotometer (Thermo Fisher Scientific, Waltham, MA). RNA integrity was assessed using the Agilent Bioanalyzer 2100 system (Agilent Technologies, Santa Clara, CA).

### Library preparation for transcriptome sequencing

A total amount of 1 µg RNA per sample was used as input material for the RNA sample preparations. Sequencing libraries were generated using NEBNext Ultra RNA Library Prep Kit for Illumina (NEB, Ipswich, MA, USA) following the manufacturer’s recommendations. Briefly, mRNA was purified from total RNA using poly-T oligo-attached magnetic beads. Fragmentation was carried out using divalent cations under elevated temperature in NEBNext First Strand Synthesis Reaction Buffer (5X). First-strand cDNA was synthesized using random hexamer primers and M-MuLV Reverse Transcriptase (RNase H). Second-strand cDNA synthesis was subsequently performed using DNA Polymerase I and RNase H. Remaining overhangs were converted into blunt ends via exonuclease/polymerase activities. After adenylation of 3ʹ ends of DNA fragments, NEBNext Adaptors with hairpin loop structure were ligated to prepare for hybridization. To select cDNA fragments of preferentially 150–200 bp in length, the library fragments were purified with the AMPure XP system (Beckman Coulter, Beverly, MA). Then 3 µL of USER Enzyme (NEB) was used with size-selected, adaptor-ligated cDNA at 37 °C for 15 min followed by 5 min at 95 °C before polymerase chain reaction (PCR). Then PCR was performed with Phusion High-Fidelity DNA polymerase, Universal PCR primers and Index (X) Primer. At last, PCR products were purified (AMPure XP system) and library quality was assessed on the Agilent Bioanalyzer 2100 system.

### Clustering and next-generation RNA sequencing

Cluster generation and sequencing were performed by Novogene (UK) Company Limited (Cambridge, UK). The clustering of the samples was performed on a cBot Cluster Generation System using a PE Cluster kit cBot-HS (Illumina). After cluster generation, the library preparations were sequenced on the Illumina HiSeq2000 platform to generate paired-end reads whose size was paired-end 2 × 150bp reads. Raw reads in fastq format were first processed through in-house perl scripts. In this step, clean data were obtained by removing reads containing adapters, reads containing poly-N and low-quality reads. At the same time, Q20, Q30, GC content and sequence duplication levels of the clean data were calculated. All the downstream analyses were based on high-quality clean data (see Table 2).

**Table 2.** Summary statistics of the RNA quality and sequencing results.

Average RIN	6.6
Clean reads	216 million
No. of transcripts	94 179
No. of unigenes	39 253
Average of read mapped rate	84.03 %
Transcripts N50 (bp)	1897
Unigenes N50 (bp)	1854
Q30 (%)	95.24
GC content (%)	43.31

### De novo assembly and gene functional annotation

*De novo* transcriptome assembly was made up by Trinity software (2.6.6 version) with min_Kmer_Cov = 3 and min_glue = 4 (Grabherr *et al.* 2013). Hierarchical Clustering was carried out by Corset (4.6 version) to remove redundancy (parameter – m 10) so that the longest transcript of each cluster has been selected as unigene (Davidson and Oshlack 2014). The assembly assessment and gene prediction were performed by Benchmarking Universal Single-Copy Orthologous (BUSCO software, 3.0.2 version; Simão *et al.* 2015), whereas the unigene functional annotations were obtained by exploiting seven different databases: National Centre for Biotechnology Information (NCBI), non-redundant protein sequences (Nr, Diamond software, 0.8.22 version, e-value threshold 1e-5; Buchfink *et al.* 2014), NCBI non-redundant nucleotide sequences (Nt, NCBI blast software, 2.9.0 version, e-value threshold 1e-5), Protein family (Pfam, hmmscan software, HMMER 3.1 version, e-value threshold 0.01; Finn *et al.* 2011), Cluster of Orthologous Groups of Proteins (KOG/COG, Diamond software, 0.8.22 version, e-value threshold 1e-5; Buchfink *et al.* 2014), Swiss Prot (Diamond software, 0.8.22 version, e-value threshold 1e-5), Kyoto Encyclopaedia of Genes and Genome (KEGG, Diamond and KAAS software, 0.8.22 version, e-value threshold 1e-5; Moriya *et al.* 2007; Buchfink *et al.* 2014) and GO (blast2GO software, b2g4pipe_v2.5 version, e-value threshold 1e-6; Götz *et al.* 2008). The *L. sativa* transcriptome was submitted to NCBI (https://www.ncbi.nlm.nih.gov/geo/) accession number (GSE227491).

### Quantification of gene expression and differential expression analysis

Gene expression level was estimated by RSEM software (1.2.28 version) by mapping back each clean read onto assembled transcriptome and the read counts for each gene were then obtained from the mapping results. Furthermore, the read counts of each gene have been used as input data for DESeq2 (1.26 version, padj ≤ 0.05), to obtain differentially expressed genes (DEGs; Love *et al.* 2014). The resulting *P*-values were adjusted using the Benjamini and Hochberg’s approach for controlling the false discovery rate. The genes with an adjusted *P*-value ≤ 0.05 were assigned as differentially expressed.

### Real-time validation of selected DEG candidates using qRT-PCR

Leaf total RNA (2.5 µg) was reverse transcribed using SuperScript Vilo cDNA synthesis kit by Thermo Fischer Scientific, according to the manufacturer’s instructions. Real-time qRT-PCR was carried out for nine DEGs with PowerUp SYBR Green Master mix by Thermo Fischer Scientific. All the genes have been normalized with the endogenous reference gene encoding the ribosomal RNA small subunit methyltransferase (LOC111912865) and the fold change was calculated by the 2−^∆∆CT^ method (Livak and Schmittgen 2001). The sequences of primers used for real-time PCR are provided in Supporting Information—**Table S1**.

### KEGG, GO, Mapman and iTAK enrichment analysis

For enrichment analysis, all the DEGs were submitted to KOBAS software (version 3.0, corrected *P*-value ≤ 0.05) to identify the significantly enriched pathways in the KEGG database (Mao *et al.* 2005). The GO functional enrichment analysis of the DEGs was implemented by using either blast2go (b2g4pipe, version v2.5, e-value threshold 1e−6) or GOSeq (version 1.32.0, corrected *P*-value ≤ 0.05) softwares. Moreover, a pathway analysis was conducted using MapMan3.6.0RC1 (https://mapman.gabipd.org/). All the unigenes were annotated and mapped using Mercator4 V2.0, an online tool of MapMan (https://www.plabipd.de/portal/mercator4) which accurately assigns hierarchal ontology providing a visual representation of genes in different plant processes. The significant DEGs (*P*_adj_ ≤ 0.05), with the corresponding log_2_fold change values, were used as dataset to align with the Mercator map. Furthermore, we focused on those clusters showing a threshold of ±1.5 log_2_fold change. For each cluster, sequence alignment has been performed and the score of these alignments (*L. sativa*, 100% identity and e-value = 0) provided clear indications of the cluster identity. iTAK (hmmerscan software) tool was used to identify the transcription factor (TF) families among DEGs (Pérez-Rodríguez *et al.* 2009; Jin *et al.* 2014). Furthermore, to identify the core gene set responding to microalgal treatments, the significant DEGs (*P*_adj_ ≤ 0.05) belonging to both *Ls*Cv versus *Ls*CK and *Ls*Sq versus *Ls*CK comparisons (threshold of ±1.50 log_2_fold change) were retrieved and merged in a list of genes responding to both algal treatments and deregulated in the same direction (up- or down-regulated). All these genes were subjected to GO and Mapman enrichment analysis as described above.

## Results

### Effect of microalgae extracts upon lettuce seedling morpho-biometric parameters

As shown in Table 1, the application of microalgae extracts positively influenced the seedling morphological traits. In detail, the application of *C. vulgaris* and *S. quadricauda* extracts reduced the root/shoot FW ratios, indicating that better-growing conditions have been reached (Bohne *et al.*, 2009). *C. vulgaris* and *S. quadricauda* extracts did not affect both the shoot and root FW/DW ratios, thus suggesting that the treatments determined a biomass accumulation in terms of dry matter at a comparable extent to the control conditions (Table 1). As reported in Table 1, the RGR, whose value increases as function of an ameliorated nutritional status of the plant (Gent 2017), resulted to be higher in treated samples than that calculated for control seedlings. In particular, the RGR in *Ls*Cv samples was higher than that measured in the *Ls*Sq thesis, thus suggesting that *C. vulgaris* extract could exert a more pronounced biostimulant effect on lettuce seedlings.

**Table 1.** Growth parameters of lettuce seedlings subjected to *C. vulgaris* (*Ls*Cv) and *S. quadricauda* (*Ls*Sq) treatments (*Ls*CK: control; FW: fresh weight; DW: dry weight; RGR: relative growth rate). Different letters indicate significance according to Fisher’s protected LSD test (*P* = 0.05); *, **, and ***: significance of *P* ≤ 0.05, 0.01, and 0.001, respectively. ns: not significant.

Sample	Root/shoot FW ratio	Root/shoot DW ratio	Shoot FW/DW	Root FW/DW	RGR
Control	0.26a	0.39a	16.59a	10.60a	0.0068c
*Ls*Cv	0.20b	0.35a	16.67a	9.98a	0.0255a
*Ls*Sq	0.13c	0.27a	17.61a	9.80a	0.0163b
	**	ns	ns	ns	***

### Transcript assembly and annotation

In this study, a comprehensive identification of the transcriptional response of *L. sativa* seedlings to *C. vulgaris* and *S. quadricauda* extracts was conducted by applying a RNASeq approach. The quality of RNA was assessed before the preparation of the libraries by the RNA integrity number (RIN) measurement. The mean RIN value was 6.6, indicating that a low level of RNA degradation occurred, thus all samples were adequate for further processing and sequencing (Table 2). After library sequencing, we filtered the raw reads to remove the adapter-based or poor-quality reads, obtaining a total of 216 million clean reads (Table 2), representing the 98.02% of the total reads. Downstream analysis was further performed on about 36 million reads (10.82 Gb per sample), showing Q30 and GC content equal to 95.24% and 43.31%, respectively (Table 2). The clean read *de novo* assembly yielded 94,179 transcripts and 39,253 unigenes with N50 length of 1897 bp and 1854 bp, respectively (Table 2), consistent with previously reported N50 values (Sicilia *et al.* 2019, 2020; Zhang *et al.* 2019) and indicating that a good coverage of the transcriptome has been achieved. To assess assembly consistency, filtered unique reads were mapped to the reconstructed transcriptome and the average read mapping rate using bowtie2 alignment software was equal to 84.03 % (Table 2). The completeness of the assembled transcriptome was evaluated by comparing it to the set of Embryophyta genes using the BUSCO quality assessment tool coupled with the OrthoDB (9.0 version) database of orthologs (Simão *et al.* 2015). The quality of the *L. sativa* leaf transcriptome was comparable to those of the majority of transcriptome assemblies listed in Simão *et al.* (2015). Among the searched 1440 BUSCO groups, 76.25 % (1098 BUSCOs) was complete (1046 single-copy orthologs and 52 duplicated), 10.9 % (157 BUSCOs) was represented by fragments and 12.9 % (185 BUSCOs) was missing. In addition, both transcript and unigene length distributions were reported [see Supporting Information**—Fig. S1]**.

Functional annotation of the lettuce unigenes was conducted by performing BLAST searches against public databases, such as the National Center for Biotechnology Information (NCBI), Protein Family (Pfam), Protein Ortholog Group Clusters (KOG/COG), SwissProt, Ortholog Database (KO), Gene Ontology (GO) (Table 3). A total of 33 819 unigenes were annotated in at least one database, and the frequency of unigenes annotated in at least one searched database was 86.15 %. Among them, 29 515 (75.19 %) and 30 498 (77.69 %) unigenes showed identity with the sequences in the Nr and Nt databases, respectively. The distributions of unigene homologous to the sequences in the KO, SwissProt, Pfam, GO, and KEGG databases were 25.24 %, 56.84 %, 52.81 %, 52.81 % and 18.77 %, respectively (Table 3).

**Table 3.** The number and percentage of successful annotated genes.

Database	Number of unigenes	Percentage (%)
Annotated in NR	29 515	75.19
Annotated in NT	30 498	77.69
Annotated in KO	9908	25.24
Annotated in SwissProt	22 314	56.84
Annotated in PFAM	20 733	52.81
Annotated in GO	20 731	52.81
Annotated in KOG	7369	18.77
Annotated in at least one database	33 819	86.15

### Identification of differentially expressed genes

The characterization of leaf *L. sativa* transcriptome was carried out by the identification of those unigenes whose expression level changed upon microalgal extract treatments. Based on the experimental design, a total of 16,754 DEGs were observed from all the comparisons. Among them, 3254 up-regulated genes and 3943 down-regulated genes were detected in the *Ls*Cv versus *Ls*CK (samples treated with *C. vulgaris* vs. untreated samples), whereas in the case of *Ls*Sq versus *Ls*CK (samples treated with *S. quadricauda* vs. untreated samples) a total of 2773 up-regulated genes and 4345 down-regulated genes were identified (Table 4). Table 4 also reports the number of deregulated genes in the *Ls*Cv versus *Ls*Sq comparison (samples treated with *C. vulgaris* vs samples treated with *S. quadricauda*). A total of 2439 DEGs were in this last comparison, 1374 of them resulted up-regulated and 1065 down-regulated, thus indicating that a distinct response was induced upon lettuce seedlings in a species-specific manner by the two algal extracts under investigation. However, transcripts belonging to both the *Ls*Cv versus *Ls*CK and *Ls*Sq versus *Ls*CK comparisons and showing the same direction of deregulation (up- or down-regulated) were retrieved and included in a list representing the core gene set that responded to treatments in a microalgal species-independent manner [see Supporting Information**—Table S2]**. The list includes 1330 clusters, 1184 of which were down-regulated and 146 up-regulated, suggesting that the effects of algal extracts mainly involve the repression of a high number of lettuce genes.

**Table 4.** DEG number of different comparisons under microalgae treatments.

	Up-regulated	Down-regulated	Total DEGs
LsCv vs LsCK	3254	3943	7197
LsSq vs LsCK	2773	4345	7118
LsCv vs LsSq	1374	1065	2439
Total DEGs	7401	9353	16 754

### Validation of RNAseq experiments by real-time PCR

The validation of gene expression levels for nine selected DEG candidates was carried out by quantitative real-time PCR (coefficient of determination *R*^2^ = 0.91), indicating the reliability of RNA Seq in the quantification of gene expression [see Supporting Information**—Fig. S2]**. In addition, the selected genes could also constitute useful markers of microalgal extract response in lettuce.

### GO and Mapman enrichment analysis of the core gene set deregulated in algal species-independent manner

**Figure 1.**
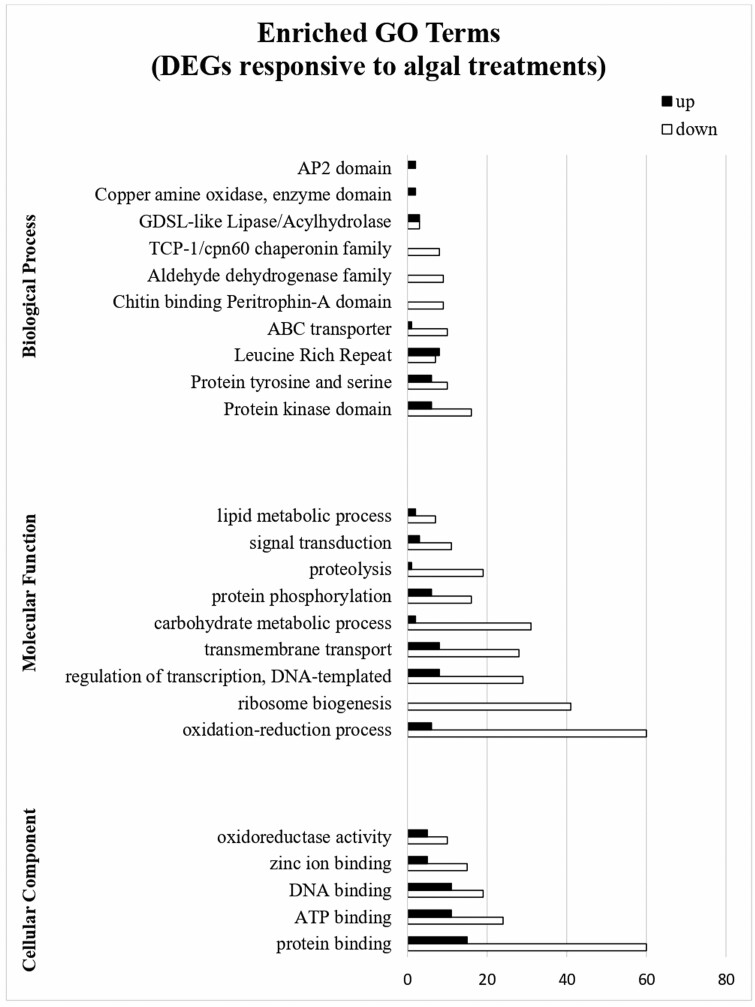
GO enrichment analysis for the DEGs in common between the *Ls*Cv vs *Ls*CK and *Ls*Sq vs *Ls*CK comparisons in *L. sativa*. The Y-axis indicates the subcategories, and the X-axis indicates the numbers related to the total number of GO terms. BP, biological process; MF, molecular functions; CC, cellular components.

The GO functional enrichment analysis of those clusters belonging to both the *Ls*Cv versus *Ls*CK and *Ls*Sq versus *Ls*CK comparisons and showing the same direction of deregulation (146 up-regulated and 1184 down-regulated) is shown in Fig. 1. ‘Protein kinase domain’ (GO:0051603) (6 up- and 16 down-regulated genes), ‘Protein tyrosine and serine’ (GO:0016310) (6 up- and 10 down-regulated genes), ‘Leucine rich repeat’ (GO:0006913) (8 up- and 7 down-regulated genes) and ‘ABC transporter’ (GO:0006810) (1 up- and 10 down-regulated genes) are the most enriched GO terms found in the Biological Process (BP) category. ‘Oxidation-reduction process’ (GO:0016702) (6 up- and 60 down-regulated genes), ‘ribosome biogenesis’ (GO:0042254) (0 up- and 41 down-regulated genes), ‘regulation of transcription, DNA-templated’ (GO:0006355) (8 up- and 29 down-regulated genes) and ‘transmembrane transport’ (GO:0055085) (8 up- and 28 down-regulated genes) are the most enriched GO terms in the Molecular Function (MF) category. Among the DEGs belonging to the Cellular Component (CC) category, the most represented GO terms are ‘protein binding’ (GO:0005515) (15 up- and 60 down-regulated genes), ‘ATP binding’ (GO:0005524) (11 up- and 24 down-regulated genes) and ‘DNA binding’ (GO:0003677) (11 up- and 19 down-regulated genes). All the significant DEGs were also analysed with the Mapman 3.6.0RC1 software and ‘protein homeostasis’ (33 DEGs, 4 up- and 29 down-regulated), ‘lipid metabolism’ (18 DEGs, 3 up- and 15 down-regulated), ‘phytohormone’ (9 DEGs, 5 up- and 4 down-regulated) and ‘amino acid metabolism’ (6 DEGs, 0 up- and 6 down-regulated) are the categories mainly deregulated by the algal treatments [see Supporting Information**—Table S3]**.

### Functional classification of DEGs

**Figure 2.**
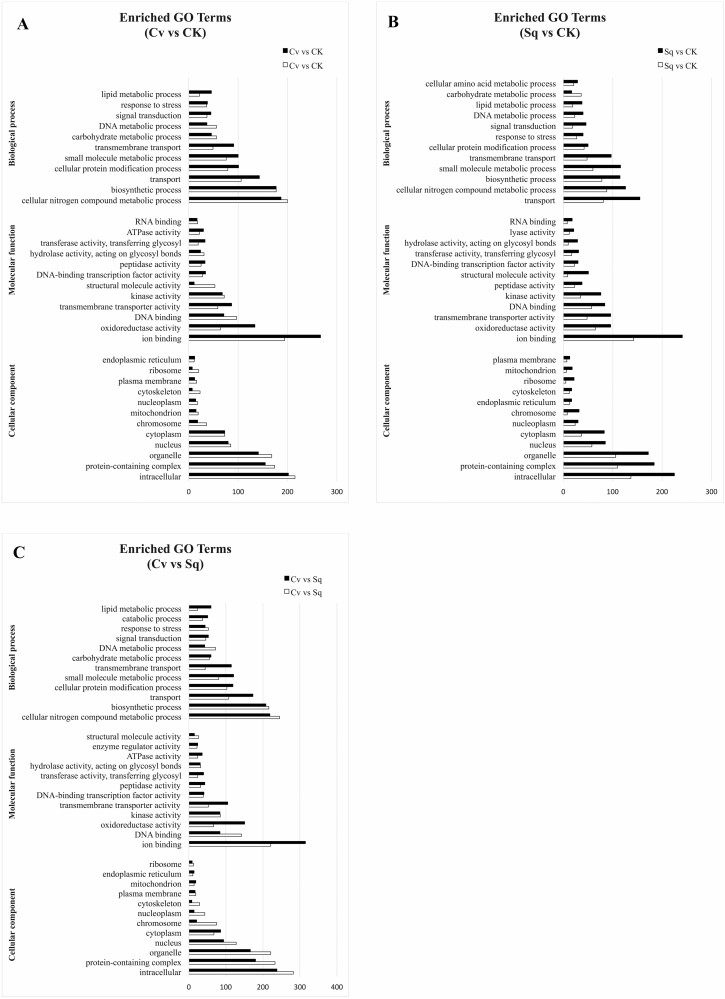
GO enrichment analysis for the DEGs in *L. sativa*. (A) *Ls*Cv vs *Ls*CK. (B) *Ls*Sq vs *Ls*CK (C) LsCv vs LsSq. The Y-axis indicates the subcategories, and the X-axis indicates the numbers related to the total number of GO terms. BP, biological processes; CC, cellular components; MF, molecular functions.

Gene Ontology terms, Clusters of Orthologous Groups of proteins (KOG) classification and KEGG pathway functional enrichment were carried out to identify biological processes or pathways specifically involved in lettuce seedling response to microalgal extract treatments. Considering the *Ls*Cv vs *Ls*CK data set (Fig. 2A), ‘Cellular nitrogen compound metabolic process’ (GO:0034641) (229 up- and 227 down-regulated genes), ‘biosynthetic process’ (GO:0009058) (177 up- and 177 down-regulated genes) and ‘transport’ (GO:0006810) (143 up- and 106 down-regulated genes) are the most enriched GO terms found in the BP category. ‘Ion binding’ (GO:0043167) (267 up- and 194 down-regulated genes), ‘oxidoreductase activity’ (GO:0016491) (134 up- and 64 down-regulated genes) and ‘DNA binding’ (GO:0003677) (71 up- and 97 down-regulated genes) are the most enriched GO terms in the MF category. Among the DEGs belonging to the CC category, the most represented GO terms are ‘intracellular’ (GO:0005622) (202 up- and 215 down-regulated genes), ‘protein-containing complex’ (GO:0032991) (155 up- and 174 down-regulated genes) and ‘organelle’ (GO:0043226) (141 up- and 168 down-regulated genes).

Among the DEGs belonging to *Ls*Sq versus *Ls*CK dataset, ‘Transport’ (GO:0006810) (155 up- and 81 down-regulated genes), ‘cellular nitrogen compound metabolic process’ (GO:0034641) (126 up- and 88 down-regulated genes), ‘biosynthetic process’ (GO:0009058) (115 up- and 78 down-regulated genes) and ‘small molecule metabolic process’ (GO:0044281) (116 up- and 80 down-regulated genes) are the most represented GO terms identified in the BP category. ‘Ion binding’ (GO:0043167) (241 up- and 142 down-regulated genes), ‘oxidoreductase activity’ (GO:0016491) (96 up- and 65 down-regulated genes), and ‘transmembrane transport activity’ (GO:0022857) (96 up- and 48 down-regulated genes) are over-represented in the MF category (Fig. 2B). In the CC category, ‘intracellular’ (GO:0005622) (225 up- and 137 down-regulated genes), ‘protein-containing complex’ (GO:0032991) (184 up- and 110 down-regulated genes) and organelle (GO:0043226) (172 up- and 106 down-regulated genes) were highly represented. A similar trend characterized also the comparison *Ls*Cv versus *Ls*Sq since the same categories are represented (Fig. 2C).

**Figure 3.**
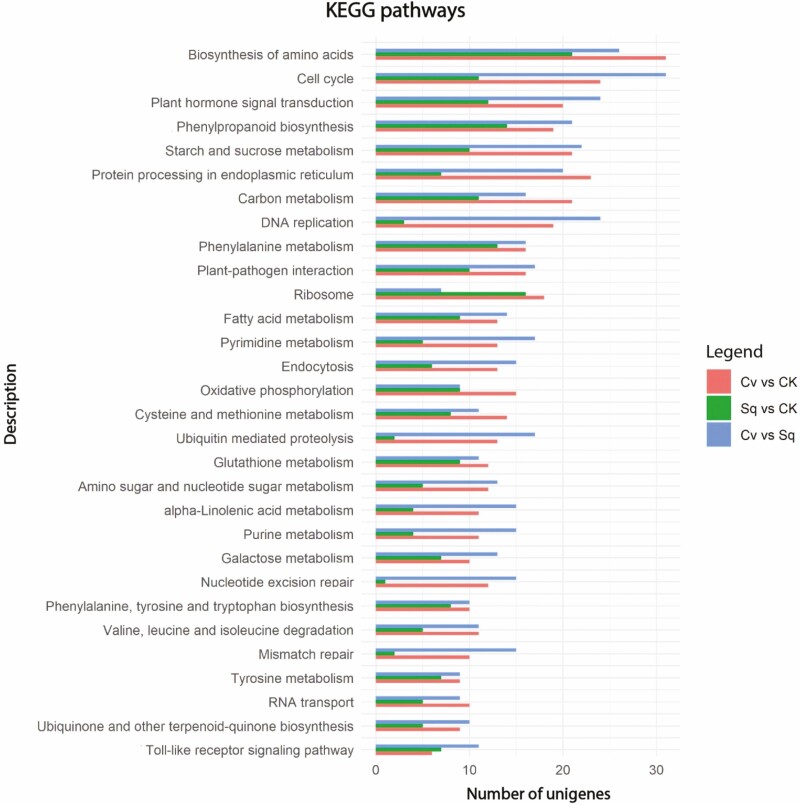
The main KEGG biological pathways for the DEGs in lettuce leaf transcriptome. The Y-axis indicates the KEGG categories, and the X-axis indicates the numbers of unigenes.

To predict and classify possible functions, all the 39,253 unigenes were aligned to the KOG database and were assigned to the KOG categories [see Supporting Information**—Fig. S3]**. Among the KOG categories, the cluster for ‘posttranslational modification, protein turnover and chaperones’ (12.30 %) represented the largest group, followed by ‘general function prediction only’ (11.40 %) and ‘translation, ribosomal structure and biogenesis’ (10.46 %) [see Supporting Information**—Fig. S3]**. To identify biological pathways activated in response to microalgae extracts, DEGs were also mapped onto the KEGG database. Figure 3 shows the main metabolic pathways sorted by the decreasing gene number involved in each pathway in relation to all the comparisons under investigation (*Ls*Cv vs. *Ls*CK, *Ls*Sq vs. *Ls*CK and *Ls*Cv vs. *Ls*Sq). Interestingly, the results indicate that the maximum number of DEGs were observed in the ‘biosynthesis of amino acids’, ‘cell cycle’, ‘plant hormone signal transduction’ and ‘starch and sucrose metabolism’, indicating that a deep metabolic reprogramming occurred in presence of the microalgal extracts (Fig. 3). The re-modulation of the metabolic machinery is also supported by the involvement of other important pathways, such as ‘carbon metabolism’ and ‘phenylpropanoid biosynthesis’, which play a pivotal role both in primary and secondary metabolisms thus confirming our previous results (Puglisi *et al.* 2020*b*). It is also worth to note that among the most enriched metabolic pathways, ‘protein processing in endoplasmic reticulum’, ‘ribosome’ and ‘RNA transport’ which are involved in mRNA translation to a polypeptide chain were deeply regulated by microalgae extracts (Fig. 3).

### Comprehensive analysis of the main pathways induced by microalgal extracts

**Figure 4.**
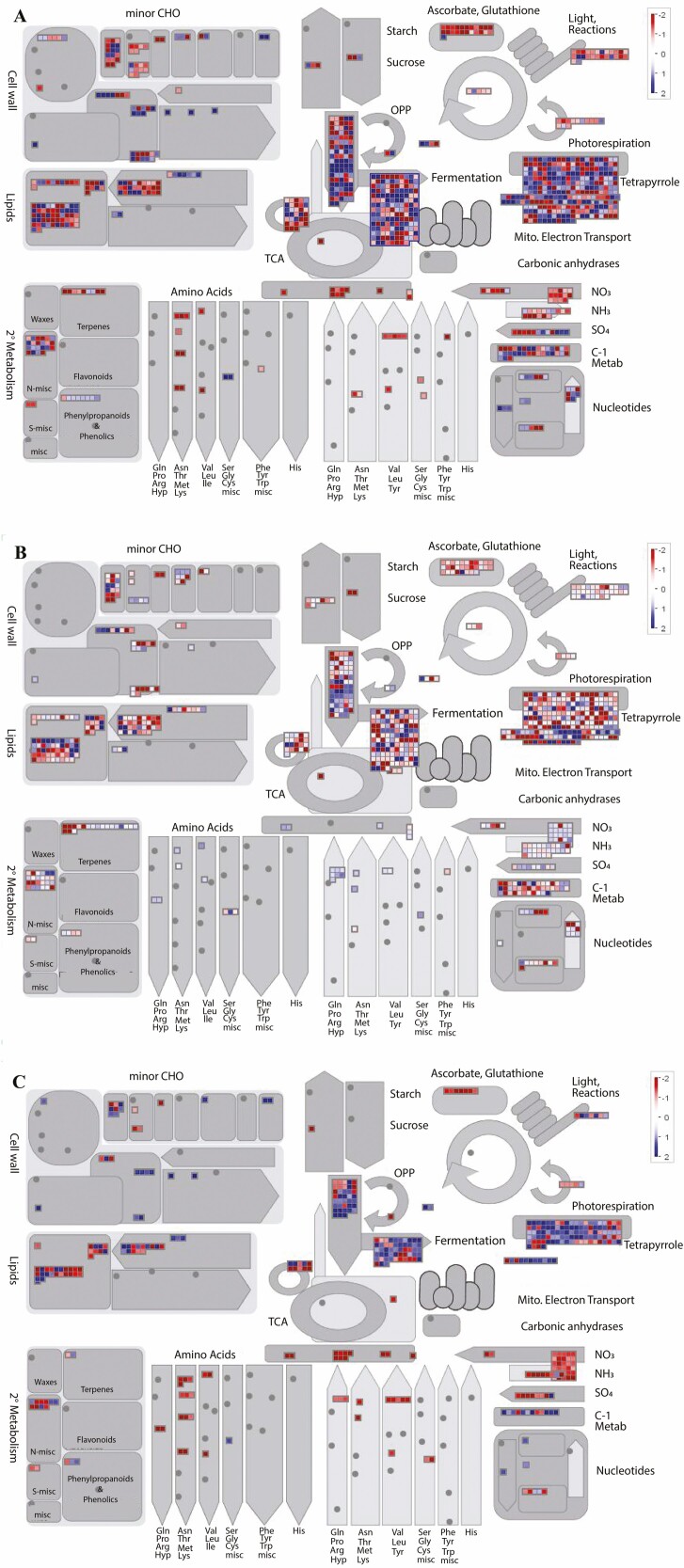
MapMan analysis of differentially expressed genes in *L. sativa*. (A) *Ls*Cv vs *Ls*CK. (B) *Ls*Sq vs *Ls*CK. (C) *Ls*Cv vs *Ls*Sq. Blue spots represent up-regulated genes and red spots represent down-regulated genes.

To obtain a complete picture of the metabolic changes occurring in lettuce seedlings treated by microalgae extracts, all the significant DEGs were mapped to the Mapman 3.6.0RC1 software. As shown in Fig. 4, several genes resulted deregulated (activated or inhibited) by either algal treatments (Fig. 4A and B). However, a shaper response was obtained in the *Ls*Cv versus *Ls*CK comparison (Fig. 4A) than in the other comparisons (Fig. 4B and C) thus indicating that the global response of lettuce to *C. vulgaris* was more pronounced than the response to *S. quadricauda*. Accordingly, to decipher the lettuce leaf response to algal treatments, we filtered the significant Mapman enriched DEGs by applying a ±1.5 log_2_fold change filter and counted the DEGs belonging to each category. As shown in Supporting Information**—Table S4**, ‘protein homeostasis’ (253 DEGs), ‘phytohormone’ (148 DEGs), ‘lipid metabolism’ (140 DEGs) and ‘amino acid metabolism’ (95 DEGs) are the categories mainly deregulated by the algal treatments. In the ‘protein homeostasis’ category, the up-regulation of several genes responsible for protein turnover was observed, including those encoding for chaperone, ubiquitin ligase, serine carboxypeptidase and proteases thus indicating that a strong rearrangement of the protein metabolism is strictly induced in response to microalgae extract treatment (data not shown). A second group of categories includes those clusters counting between 52 and 25 DEGs. Among these categories, ‘cell division’ (46 DEGs) and ‘cell wall organization’ (25 DEGs) are probably related with higher seedling growth induced by the algal treatment (Puglisi *et al.* 2022). In addition, the ‘redox homeostasis’ category (32 DEGs) lists a group of genes encoding glutathione peroxidases and glutathione transferases confirming their role in protecting plant cells both in normal and stressful conditions (data not shown; Lo Piero *et al.* 2010; Puglisi *et al.* 2013). Finally, a third group comprises categories including from 18 to 3 DEGs such as ‘protein translocation’ (18 DEGs) and ‘photosynthesis’ (10 DEGs) [see Supporting Information**—Table S4]**.

### Dissection of the ‘phytohormone’ and ‘transcription factor’ categories

**Figure 5.**
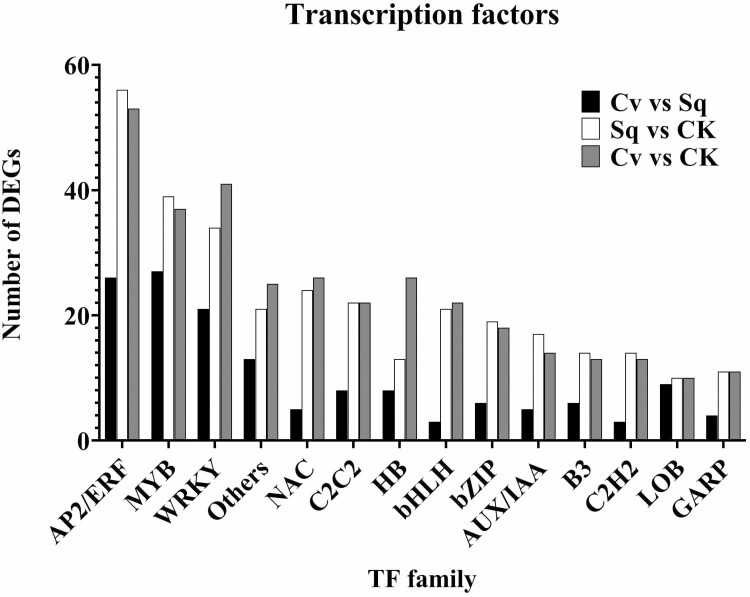
Number of DEGs encoding for TFs found in the three comparisons (*Ls*Cv vs *Ls*CK, *Ls*Sq vs *Ls*CK and *Ls*Cv vs *Ls*Sq).

Considering their main role in transcriptome reprogramming, we further dissected both the ‘phytohormones’ (Tables 5 and 6) and ‘transcription factor’ categories (Fig. 5). Table 5 includes the DEGs of the ‘phytohormone’ category that have been found specifically deregulated *by C. vulgaris* (*Ls*Cv vs. *Ls*Sq comparison) and reports the log_2_fold change of each deregulated cluster. The gene encoding the isopentenyltransferase (IPT), responsible for the rate-limiting step of cytokinin biosynthesis, was drastically down-regulated in the *Ls*Cv versus *Ls*Sq (−8.50 log_2_fold change), and concordantly, the cytokinin independent 1 histidine kinase, an activator of the cytokinin signalling pathway, was down-regulated. In addition, cytokinin phosphoribohydrolase (LOG) encoding gene catalysing the direct activation pattern was also down-regulated. Zeatin-type-cytokinin synthase (CYP735A), involved in later steps of cytokinin biosynthesis, was found up-regulated in *C. vulgaris* treated samples in comparison with those seedlings treated with *S. quadricauda*. However, the gene encoding the zeatin O-glucosyltransferase (ZOG) which glycosylates cytokinins leading to the cytokinin forms with reduced biological activity, was also up-regulated indicating that in *C. vulgaris* treated samples these hormones and the induction of the related signal cascade are repressed with respect of the *S. quadricauda* treated seedlings (Table 5). Clusters encoding the ligands negatively influencing stomatal density (EPF/EPFL, epidermal patterning factor; Rychel *et al.* 2010) were found down-regulated in the *C. vulgaris* treated samples. Another group of clusters related to cell proliferation was also found down-regulated in the *Ls*Cv versus *Ls*Sq comparison (Table 5): (i) the TDIF (Tracheary element Differentiation Inhibitory Factor) peptide and the TDR/PXY (TDIF receptor/Phloem intercalated with Xylem) membrane protein kinase, promoting the proliferation of procambial cells and suppressing their xylem differentiation (Hirakawa *et al.* 2010), (ii) the EMS1 (EXCESS MICROSPOROCYTES1) LRR-RLK and its small protein-ligand TPD1 (TAPETUM DETERMINANT1), that play a fundamental role in somatic and reproductive cell differentiation during early anther development in *Arabidopsis* (Li *et al.* 2017), and (iii) the phytosulfokine receptor which regulates a signalling cascade involved in plant cell differentiation, organogenesis, somatic embryogenesis, cellular proliferation and plant growth.

**Table 5.** DEGs listed in the ‘Phytohormone’ category specifically deregulated in *Ls*Cv versus *Ls*Sq.

Cluster ID	Database description	log_2_fold change LsCv vs LsSq
9839.0	*IP-type-cytokinin synthase* (*IPT3*)	−8.50
15809.3160	*Cytokinin signalling pathway activator* (*CKI1*)	−2.12
15809.14844	*Cytokinin phosphoribohydrolase* (*LOG3*)	−1.45
16226.0	*Cytokinin hydroxylase*	+2.86
3690.0	*Zeatin O-glucosyltransferase* (*ZOG*)	+3.20
15809.11964	*EPF/EPFL epidermal patterning factor*	−2.26
11176.0	*TDL-peptide receptor* (*EMS1/MSP1*)	−2.03
6384.0	*Regulatory protein kinase* (*PINOID2*) *of auxin transport*	−1.76
15809.1007	*TDIF-peptide receptor* (*PXY*)	−1.69
10341.0	*Pythosulfokine peptide receptor* (*PSKR1*)	−1.61
14797.0	*Flavin-dependent monooxygenase* (*YUCCA10*)	+2.42
8784.0	*Flavin-dependent monooxygenase* (*YUCCA5*)	+2.52

**Table 6.** DEGs listed in the ‘Phytohormone’ category in common between *Ls*Cv vs *Ls*CK and *Ls*Sq vs *Ls*CK.

Cluster ID	Database description	log_2_fold change LsCv vs LsCK	log_2_fold change LsSq vs LsCK
8434.0	*Transcriptional repressor* (*IAA27/AUX*)	−4.12	−2.29
17059.0	*CIF precursor polypeptide*	−3.17	−2.55
5788.0	*Transcriptional repressor* (*IAA17/AUX*)	−3.09	−1.93
15809.12001	*RALF-peptide receptor* (*CrRLK1L*) – *THESEUS*	−2.48	−2.20
15809.3701	*Brassinosteroid signalling protein kinase*	−2.18	−2.02
18385.0	*RALF-peptide receptor* (*CrRLK1L*) – *FERONIA*	+1.99	+1.78
14748.0	*B-type ARR response activator of cytokinin*	+2.70	+2.32
15809.8656	*PYL/RCAR abscisic acid receptor PYL4-like*	+2.90	+2.34
4711.0	*Auxin efflux transporter* (*PILS7*)	+4.30	+2.79
6996.0	*PNP precursor polypeptide* (*EG45-like*)	+11.00	+6.61

Interestingly, two clusters encoding indole-3-pyruvate monooxygenase involved in auxin biosynthesis during embryogenesis and seedling development (Zhao 2010) were up-regulated in *Ls*Cs vs *Ls*Sq comparison suggesting that auxin is strongly implicated in the response of lettuce towards *C. vulgaris* treatment. Moreover, the down-regulation of regulatory protein kinase (PINOID) of auxin transport functioning as a positive regulator of polar auxin transport (Benjamins *et al.* 2001) indicates that the fine-tuning of polar auxin transport during organ formation in response to local auxin concentrations is affected in *C. vulgaris* treated samples.

Finally, in Table 6 the DEGs in common between the *Ls*Cv versus *Ls*CK and *Ls*Sq versus *Ls*CK comparisons related to the ‘phytohormone’ category are reported. All of them are subjected to the same de-regulation type (up- or down-regulated) in both comparisons, but in all cases, the extent of gene de-regulation is higher in *Ls*Cv versus *Ls*CK than in *Ls*Sq versus *Ls*CK (Table 6) as encountered by MapMan analysis (Fig. 4). Among the down-regulated genes we enumerate the transcriptional repressor (IAA/AUX) that represses the expression of primary/early auxin response genes (Tiwari *et al.* 2004), confirming the crucial role of auxin signal transduction during algal treatment. Moreover, the CASPARIAN STRIP INTEGRITY FACTOR (CIF) that triggers the spatially precise deposition of designated cell wall components, enabling plants to establish transcellular barrier networks correctly (Table 6). RALF-peptide receptor (*Catharanthus roseus* receptor-like kinase 1-like, CrRLK1L)—THESEUS, previously shown in *Arabidopsis* to trigger growth inhibition and defense responses upon perturbation of the cell wall (Gonneau *et al.* 2018) was also down-regulated in both comparisons (Table 6). Among the up-regulated genes, we found an essential regulator of plant stress responses, RALF-peptide receptor (CrRLK1L)—FERONIA, and the B-type ARR response regulator of cytokinin initiating both the transcriptional response to cytokinin and a negative feedback loop that desensitizes the plant to cytokinin (Zubo *et al.* 2020). (PYR/PYL/RCAR) receptors, responsible for the regulation of the ABA signalling pathway, PIN-LIKES (PILS) proteins contributing to intracellular auxin homeostasis (Zhao *et al.* 2021) and Plant Natriuretic Peptides (PNPs) which have an important and systemic role in plant growth and homeostasis (Morse *et al.* 2004) were among the most induced genes under algal treatments. Interestingly, the PILS protein-encoding gene, identified to be putative auxin carrier at the endoplasmic reticulum (ER) and control intracellular auxin accumulation (Zhao *et al.* 2021), was highly up-regulated in algal-treated samples.

Figure 5 categorizes the DEGs encoding TFs in the three comparisons (*Ls*Cv vs. *Ls*CK, *Ls*Sq vs. *Ls*CK and *Ls*Cv vs. *Ls*Sq). Overall, for each considered family, the highest number of deregulated TFs was encountered within the *Ls*Cv versus *Ls*CK samples, and AP2/ERF, WRKY, MYB and NAC TF are numerically the most represented families.

## Discussion

The use of plant biostimulants as recent eco-friendly approach to promote crop development has caught the interest of researchers due to the variety of ways in which they can improve plant fitness. One of the most promising classes of biostimulants is represented by microalgae extracts containing a plethora of bioactive compounds whose variegated composition could be responsible for the wide range of biological effects exerted on different crops (Deolu-Ajayi *et al.* 2022). Several manuscripts have been recently published concerning the potential and advantages of using microalgae extracts as biostimulants, especially in undesired conditions (Chiaiese *et al.* 2018; González-Morales *et al.* 2021; Deolu-Ajayi *et al.* 2022; Franzoni *et al.* 2022). Overall, they report that the beneficial effects of the algal extracts pass through changes of morphological, physiological, biochemical, epigenomic, proteomic and transcriptomic nature. However, the reprogramming of gene expression can be considered the first step to generate further changes at molecular levels, and for this reason, transcriptomic analysis via RNAseq might be considered the analysis of choice to encompass the interaction between plant and biostimulant extracts. Recently, the transcriptomics of plant biostimulation under stressful conditions has been reviewed revealing that *Ascophyllum nodosum* seaweed extract is widely applied, especially upon leaves of *Arabidopsis* and tomato (González-Morales *et al.* 2021). Interestingly, the transcriptomic data indicated that *A. nodosum* seaweed extract promotes *A. thaliana* seedlings’ growth as well as the induction of genes involved in abiotic stress (Goñi *et al.* 2016).

Lettuce (*L. sativa*) is one of the most important vegetable crops grown in the Mediterranean region where saline water is frequently used for irrigation (Lucini *et al.* 2015). Thus, with a view to reduce the use of chemical fertilizers and replacing them with environment-friendly compounds, our previous works were aimed at verifying the influence of both *C. vulgaris* and *S. quadricauda* extracts upon lettuce morpho-biometric parameters and the related biochemical response (Puglisi *et al.* 2020*b*, 2022). To elucidate the response of lettuce to microalgae extracts, in this work, we sequenced and *de novo* assembled the *L. sativa* leaf transcriptome to identify differential gene expression, BPs, metabolic pathways, and molecular markers. Our results indicated that the core gene set that responded to microalgal treatments in a species-independent manner includes 1330 clusters, 1184 of which were down-regulated and 146 up-regulated, clearly suggesting that the repression of gene expression is the main effect of algal treatment associable with the stimulating activity upon lettuce seedlings. However, although the total DEGs number between *Ls*Cv versus *Ls*CK and *Ls*Sq versus *Ls*CK comparisons was similar, our results suggested that the extent of transcriptome reprogramming between the treatments under investigation was qualitatively and quantitatively different (Tables 5 and 6). In particular, we enumerated 2439 DEGs specifically deregulated on the basis of the applied algal species (*Ls*Cv vs. *Ls*Sq comparison); this result was also confirmed by both Mapman analysis (Fig. 4), which indicated that a more pronounced response was achieved using *C. vulgaris* extract, and by the RGR values which resulted higher in seedlings treated with *C. vulgaris* extract (Table 1). The analysis of biological pathways provided a comprehensive representation of the most relevant metabolic pathways reprogrammed in lettuce upon algal treatments. Among the most enriched KEGG pathways were ‘biosynthesis of amino acids’ and ‘plant hormone signal transduction’ suggesting a key role of algal extract in inducing a deep rearrangement of both hormone biosynthesis, often starting from amino acids functioning as substrates, and the related signal transduction. The analysis of the ‘Phytohormone’ category clearly indicated that in *C. vulgaris* treated samples the cytokinin biosynthesis and signal transduction were strongly repressed with respect to the *S. quadricauda* treated seedlings, whereas, auxin biosynthesis and homeostasis were activated, thus suggesting that the registered beneficial effects of both algal extracts (Puglisi *et al.* 2020*b*, 2022; Santoro *et al.* 2022) pass through different metabolic pathways and processes. The fact that a group of genes involved in cell proliferation and differentiation (EPF/EPFL epidermal patterning factor TDL-peptide receptor (EMS1/MSP1), TDIF-peptide receptor, PXY) was found down-regulated in the *Ls* Cv versus *Ls*Sq comparison, that means they are induced by *S. quadricauda* treatment, corroborates this assertion. Moreover, a recent comparative analysis (bio-compounds and fatty acids) of harvested microalgal biomass indicated that *C. vulgaris* and *S. quadricauda* extracts contain a similar amount of carbohydrates (35.10 ± 1.35 and 33.98 ± 2.29 WW^−1^, respectively). However, *S. quadricauda* extract was richer in both lipids and proteins than *C. vulgaris* extract (Zhang *et al.* 2023) thus confirming their specific biological composition which can widely justify their different mode of action.

Interestingly, the lettuce response to both algal treatments involved also the deregulation of a huge number of genes encoding hormone-like compounds or molecules related to their signal transduction cascade. In particular, lettuce seedlings perceived the external signals to self-modulate BPs through members of *Catharanthus roseus* receptor-like kinase 1-like (CrRLK1L) proteins with their ligands, rapid alkalinization factor (RALF) peptides. FERONIA (FER), a CrRLK1L member, was initially reported to act as a major plant cell growth modulator in distinct tissues (Zhang *et al.* 2020). However, as the growth of plants depends on the compromise between cell wall growth and its integrity, *Catharanthus roseus* receptor-like kinase 1-like (CrRLK1L) THESEUS1 (THE1) was previously shown in *Arabidopsis* to trigger growth inhibition and defence responses upon perturbation of the cell wall. In this context, our results show that the deregulation of FERONIA and THESEUS signalling networks might be integrated to support the integrity of the cell wall with the coordination of normal morphogenesis (Zhang *et al.* 2020). Both algal extracts induced the expression of PNP precursor polypeptide at a very high level, more in *C. vulgaris* treatment than in *S. quadricauda* (log_2_fold change +11.00 and +6.61, respectively). PNPs are a class of systemically mobile molecules involved in several physiological processes ranging from the regulation of stomatal aperture, osmotic-dependent volume changes and responses to plant pathogens (Morse *et al.* 2004). Nevertheless, understanding of the molecular mechanisms by which PNPs exert their functions is limited by the lack of comprehensive studies reporting sets of proteins they interact with to modulate levels of secondary messengers. In this respect, it has been recently proposed that PNP-A and its PNP-R2 receptor may play an important role in fine-tuning plant immune responses to avoid inappropriate induction of SA-dependent death signals in cells spatially separated from infected or damaged cells, thereby minimizing tissue damage (Lee *et al.* 2020). Both algal extracts induced the deregulation of many TF families, these include TFs of the APETALA2/ETHYLENE RESPONSIVE FACTOR (AP2/ERF) family, which have an important role in the regulation of a number of stress responses. They also respond to hormones leading to increased plant survival under stressful conditions. In addition, AP2/ERFs participate in a variety of stress tolerance, allowing them to connect a stress regulatory network (Xie *et al.* 2019) by interactions and connections with major plant hormones such as ethylene (ET) and abscisic acid (ABA), gibberellins (Gas) and cytokinins (CTK).

## Conclusions

In this work, we evaluated the effect of microalgal extracts on the transcriptomic profile of lettuce leaves. Our results clearly indicate that treatment with *C. vulgaris* induced a qualitative and quantitative deeper response than that obtained using *S. quadricauda* extract. Moreover, although both treatments lead to ameliorated morpho-biometric parameters and share the deregulation of several biological patterns, the lettuce seedlings’ transcriptomic response clearly suggests that *C. vulgaris* actives both the auxin biosynthesis and transduction pathways whereas *S. quadricauda* up-regulates cytokinin biosynthesis pathway, probably because they are rich of different amount of beneficial components. Along the major phytohormones, algal treatments implicate the reprogramming of lettuce metabolic processes through the signal cascade induced by small hormone-like molecules that can act alone or by interacting with major hormones. Most of these molecules are reported to take the field to defend plants in the occurrence of either abiotic or biotic stress, strengthening the plant response against adverse external stimuli. Moreover, this observed de-regulation of genes that are generally categorized as ‘*stress-responsive genes*’, might positively influence plants by exerting a beneficial effect during growth. Consequently, our work produced a comprehensive list of genes that might be the target for genome editing with the aim to genetically improve lettuce allowing a limited or even null use of synthetic fertilizers and pesticides.

## Supporting Information

The following additional information is available in the online version of this article –

**Figure S1.** Length distribution of transcripts and unigenes.

**Figure S2.** Validation of RNAseq by RT real-time PCR.

**Figure S3.** KOG functional classification. Clusters of orthologous groups (KOG) classification. All unigenes were aligned to the KOG database to predict and classify possible functions. (A) RNA processing and modification; (B) chromatin structure and dynamics; (C) energy production and conversion; (D) cell cycle control, cell division, chromosome partitioning; ® amino acid transport and metabolism; (F) nucleotide transport and metabolism; (G) carbohydrate transport and metabolism; (H) coenzyme transport and metabolism; (I) lipid transport and metabolism; (J) transition, ribosomal structure and biogenesis; (K) transcription; (L) replication, recombination and repair; (M) cell wall/ membrane/envelope biogenesis; (N) cell motility; (O) posttranslational modification, protein turnover, chaperones; (P) inorganic ion transport and metabolism; (Q) secondary metabolites biosynthesis, transport and catabolism; ® general function prediction only; (S) function unknown; (T) signal transduction mechanisms; (U) intracellular trafficking, secretion, and vesicular transport; (V) defense mechanisms; (W) extracellular structures; (X) unnamed protein; (Y) nuclear structure; (Z) cytoskeleton.

**Table S1.** Validation of *Lactuca sativa* L. DEGs by Real-Time qRT-PCR.

**Table S2.** List of DEGs in common between *Ls*Cv vs LsCK and *Ls*Sq vs *Ls*CK comparisons.

**Table S3.** Number of core gene set deregulated by algal treatments categorized following the Mapman enrichment analysis.

**Table S4.** Number of DEGs belonging to each Mapman category.

plad043_suppl_Supplementary_Figure_S1

plad043_suppl_Supplementary_Figure_S3

plad043_suppl_Supplementary_Figure_S2_Table_S1

plad043_suppl_Supplementary_Table_S2

plad043_suppl_Supplementary_Table_S3

plad043_suppl_Supplementary_Table_S4

## Sources of Funding

None declared.

## Contributions by the Authors

Conceptualization: A.R.L.P. Methodology: A.R.L.P., D.F.S. and A.S. Investigation: D.F.S. and E.L.B. Validation: D.F.S., I.P. and A.B. Data curation: D.F.S., A.R.L.P., I.P. and A.B. Writing-original draft preparation: A.R.L.P., D.F.S., I.P. and A.S. Writing review and editing: A.R.L.P., D.F.S. and A.S. All authors have read and agreed to the published version of the manuscript.

## Data Availability

The data underlying this article are available in the article and its Supplementary Material. Sequencing results were submitted to NCBI (https://www.ncbi.nlm.nih.gov/geo/) accession number (GSE227491).

## Notes

**Phenome, Genome and Environment. Chief Editor:** Colleen Doherty
