# Supplementary figures and images for: Transcriptomic profile of lettuce seedlings (Lactuca sativa) response to microalgae extracts used as biostimulant agents
Source: AoB Plants. 2023 Jul 2;15(4):plad043. doi: 10.1093/aobpla/plad043 (PMC10332502; doi:10.1093/aobpla/plad043)

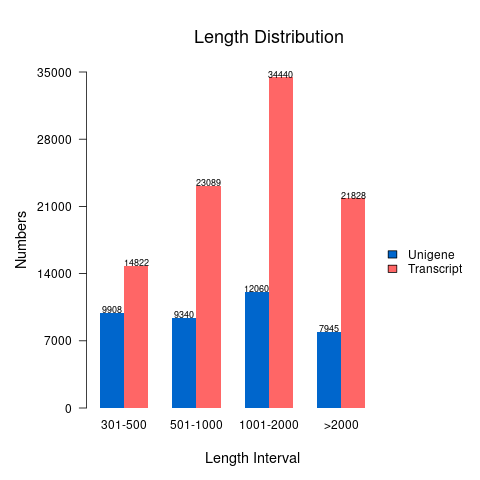


**Figure S1.** Length distribution of transcripts and Unigenes

Supplement: plad043_suppl_Supplementary_Figure_S1 [file plad043_suppl_supplementary_figure_s1.docx]
