# Supplementary material for: Transcriptomic profile of lettuce seedlings (Lactuca sativa) response to microalgae extracts used as biostimulant agents
Source: AoB Plants. 2023 Jul 2;15(4):plad043. doi: 10.1093/aobpla/plad043 (PMC10332502; doi:10.1093/aobpla/plad043)
Supplement: plad043_suppl_Supplementary_Figure_S3 [file plad043_suppl_supplementary_figure_s3.docx]

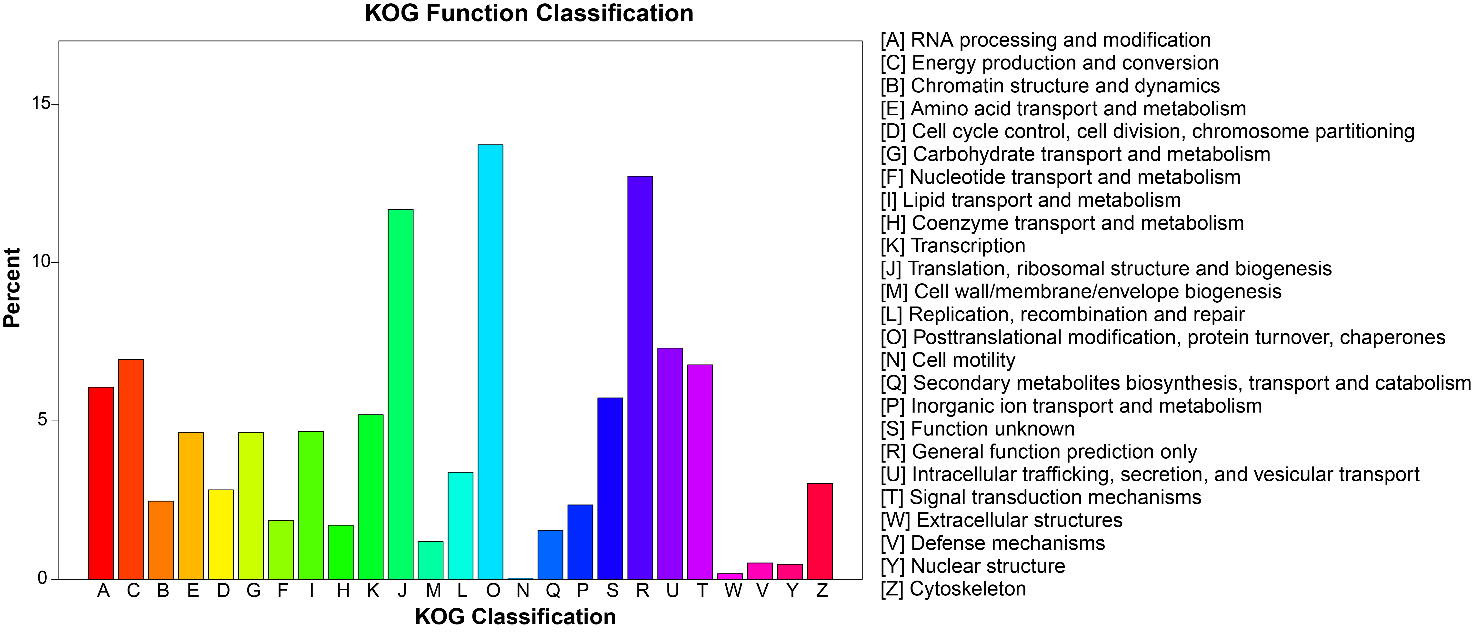


**Figure S3.** KOG functional classification. Clusters of orthologous groups (KOG) classification. All unigenes were aligned to the KOG database to predict and classify possible functions. (A) RNA processing and modification; (B) chromatin structure and dynamics; (C) energy production and conversion; (D) cell cycle control, cell division, chromosome partitioning; ® amino acid transport and metabolism; (F) nucleotide transport and metabolism; (G) carbohydrate transport and metabolism; (H) coenzyme transport and metabolism; (I) lipid transport and metabolism; (J) transition, ribosomal structure and biogenesis; (K) transcription; (L) replication, recombination and repair; (M) cell wall/ membrane/envelope biogenesis; (N) cell motility; (O) posttranslational modification, protein turnover, chaperones; (P) inorganic ion transport and metabolism; (Q) secondary metabolites biosynthesis, transport and catabolism; ® general function prediction only; (S) function unknown; (T) signal transduction mechanisms; (U) intracellular trafficking, secretion, and vesicular transport; (V) defense mechanisms; (W) extracellular structures; (X) unnamed protein; (Y) nuclear structure; (Z) cytoskeleton.
