# Supplementary material for: Transcriptomic profile of lettuce seedlings (Lactuca sativa) response to microalgae extracts used as biostimulant agents
Source: AoB Plants. 2023 Jul 2;15(4):plad043. doi: 10.1093/aobpla/plad043 (PMC10332502; doi:10.1093/aobpla/plad043)
Supplement: plad043_suppl_Supplementary_Table_S3 [file plad043_suppl_supplementary_table_s3.docx]

**Table S3. Number of core gene set deregulated by algal treatments categorized following the Mapman enrichment analysis**

|  |  |  |  |
| --- | --- | --- | --- |
| **Mapman category** | ***Up regulated*** | ***Down regulated*** | ***Total*** |
|  |  |  |  |
|  |  |  |  |
| *Protein homeostasis* | 4 | 29 | 33 |
| *Lipid metabolism* | 3 | 15 | 18 |
| *Phytohormone* | 5 | 5 | 10 |
| *Carbohydrate metabolism* | 1 | 6 | 7 |
| *Protein translocation* | 0 | 7 | 7 |
| *Secondary metabolism* | 1 | 5 | 6 |
| *Amino acid metabolism* | 0 | 6 | 6 |
| *RNA processing* | 1 | 4 | 5 |
| *Redox homeostasis* | 0 | 5 | 5 |
| *Nucleotide metabolism* | 0 | 4 | 4 |
| *Nutrient upkate* | 0 | 3 | 3 |
| *Chromatin organisation* | 0 | 1 | 1 |
| *Cellular respiration* | 0 | 1 | 1 |
| *Polyamine metabolism* | 0 | 1 | 1 |
