# Supplementary material for: Transcriptomic profile of lettuce seedlings (Lactuca sativa) response to microalgae extracts used as biostimulant agents
Source: AoB Plants. 2023 Jul 2;15(4):plad043. doi: 10.1093/aobpla/plad043 (PMC10332502; doi:10.1093/aobpla/plad043)
Supplement: plad043_suppl_Supplementary_Table_S4 [file plad043_suppl_supplementary_table_s4.docx]

**Table S4. Number of DEGs belonging to each Mapman category**

| **Category** | *LsCv vs LsCk* | *LsSq vs LsCk* | *LsCv vs LsSq* | *Total* |
| --- | --- | --- | --- | --- |
| *Protein homeostasis* | 68 | 138 | 47 | 253 |
| *Phytohormone* | 69 | 43 | 36 | 148 |
| *Lipid metabolism* | 73 | 45 | 22 | 140 |
| *Amino acid metabolism* | 58 | 18 | 19 | 95 |
| *Secondary metabolism* | 24 | 20 | 8 | 52 |
| *Cell division* | 14 | 0 | 32 | 46 |
| *Carbohydrate metabolism* | 18 | 14 | 9 | 41 |
| *Nutrient upkate* | 17 | 13 | 7 | 37 |
| *Redox homeostasis* | 13 | 11 | 8 | 32 |
| *Nucleotide metabolism* | 18 | 7 | 8 | 33 |
| *RNA processing* | 15 | 12 | 6 | 33 |
| *Chromatin organisation* | 14 | 5 | 15 | 29 |
| *Cell wall organisation* | 15 | 5 | 5 | 25 |
| *Protein translocation* | 9 | 7 | 2 | 18 |
| *DNA damage response* | 10 | 0 | 6 | 16 |
| *Photosynthesis* | 8 | 0 | 2 | 10 |
| *Coenzyme metabolism* | 5 | 2 | 2 | 9 |
| *Cellular respiration* | 0 | 3 | 1 | 4 |
| *Polyamine metabolism* | 1 | 1 | 1 | 3 |
